# Decomposing the Effects of Familiarity with Music Cues on Stride Length and Variability in Persons with Parkinson’s Disease: On the Role of Covariates

**DOI:** 10.3390/ijerph191710793

**Published:** 2022-08-30

**Authors:** Kyoung Shin Park

**Affiliations:** Department of Kinesiology, University of North Carolina at Greensboro, Greensboro, NC 27412, USA; k_park4@uncg.edu

**Keywords:** affect, gait, movement disorders, neurologic music therapy, rhythmic auditory stimulation

## Abstract

This study aimed to determine the role of cognitive and affective responses to music cues in modulating the effects of familiarity with music on stride length and stride-to-stride variability in people with Parkinson’s disease (PD). Using multilevel modeling, people with PD’s spatiotemporal gait parameters and self-reported ratings of familiarity, enjoyment, cognitive and physical demand, beats salience of music cues after each walking trial, as well as music reward, were analyzed. Our findings indicate that (1) condition-varying perceived enjoyment and beat salience are positively associated with increased stride length; (2) participants with a greater music reward for mood regulation and emotion evocation show greater stride length changes compared with those with less music reward; (3) condition-varying perceived enjoyment is positively associated with decreases in stride-to-stride variability; and (4) participants with lower cognitive demand of walking with music cues and higher beat salience show lower stride-to-stride variability compared with those with higher cognitive demand and lower beat salience. These results provide behavioral evidence of independent and interactive influences of cognitive and affective responses to music cues on spatiotemporal gait parameters in people with PD.

## 1. Introduction

Rhythmic auditory stimulation (RAS) is an application of pulsed rhythmic auditory stimuli for the facilitation of gait or gait-related aspects of movements in humans [1]. Often utilized in clinical settings, subjects are trained to synchronize rhythmic motor actions to RAS that imparts a temporal reference for movements. Because people with Parkinson’s disease (PD) often suffer from sluggish walking, short shuffling steps, hunched posture, and/or balance instability [2], RAS has been an efficacious strategy for the treatment of such motor disturbance. Compared with visual stimuli or conventional physical therapies, acute and long-term RAS interventions have provided greater benefits for the improvements of gait function in persons with PD [3,4,5]. Reported outcomes include increased gait velocity and stride length [6,7,8], reduced gait variability [9,10,11], and improved motor scores of the Unified Parkinson’s Disease Rating Scale (UPDRS; [12,13]).

Rhythmic motor entrainment, as a human’s innate predisposition [14], has been described as an underlying mechanism of RAS interventions [15]. The periodicity of auditory rhythm allows listeners to anticipate subsequent beats and then match their motor actions in time with the beats (i.e., entrainment) [15,16,17]. Gait interventions for people with PD have used varying forms of RAS, such as metronome pulses [4,6,7], subject-preferred music with lyrics [18,19,20], or instrumental music with accentuated beats [21,22,23]. Familiarity with these varying auditory stimuli could be a crucial factor for modulating entrainment. This is because the anticipation of subsequent beats, cognitive demand of entrainment, or affective engagement in rhythm could vary by the degree of familiarity with auditory stimuli. The more familiar an individual is with the rhythmic structure of stimuli, the less cognitive demand is required for motor entrainment and the more enjoyment can be evoked [24]. The effects of familiarity would be more pronounced when using music compared with a metronome, because of the complex rhythmic structure of music.

In a prior study, we demonstrated that the effects of RAS on Parkinsonian gait are modulated by familiarity with music [19]. People with mild-to-moderate PD showed greater stride length and reported greater enjoyment and beat salience when walked in sync with familiar music cues compared with unfamiliar music cues. Interestingly, acute enhancement of familiarity with unfamiliar music cues via repeated listening increased gait velocity, stride length, enjoyment, and beat salience, and reduced stride-to-stride time variability in a following session, which were not observed when walked again with familiar music cues in persons with PD. These findings are in line with existing evidence in healthy adults that familiarity with music is associated with higher ratings of pleasure and arousal responses [25], as well as the activation of limbic and reward circuits in the brain [26], and the improvement of spatiotemporal gait parameters, such as increased stride velocity, higher step–beat synchronization accuracy, and reduced stride-to-stride variability [24].

As described above, cognitive and affective engagement in RAS is evident along with motor entrainment. However, how those factors are associated with entrainment-induced changes in motor outcomes in people with PD remains unspecified. Although we reported the changes of enjoyment and beat salience along with enhanced familiarity with music and spatiotemporal gait parameters in people with PD, whether the cognitive and affective factors contribute to the refinement of Parkinsonian gait—and to what extent—needs further investigation. Therefore, a secondary data analysis was conducted to decompose the effects of familiarity with music cues on stride length and stride-to-stride variability, described in the original study [19]. This study aimed to test the hypothesis that the alterations of stride length and stride-to-stride variability by familiarity manipulation would be associated with a variant of cognitive and affective responses to RAS in persons with PD.

## 2. Materials and Methods

### 2.1. Participants

The data of twenty individuals with idiopathic PD were analyzed (age M = 68.9, SD = 5.39; 7 females; Hoehn and Yahr stage 1–3). Participants provided written informed consent that was approved by the University of Florida Institutional Review Board. Eligibility was determined based on interested individuals’ self-report or screening tests showing history of brain surgery, neurologic conditions (e.g., stroke, ataxia, tumor, etc.), orthopedic injuries, hearing problems, cognitive or affective impairments, or music anhedonia based on self-report or screening tests, as described in the original study [19]. Upon arriving at the lab, participants took their medications and then reported that they were on stable doses of orally administered levodopa. Gait trials began approximately an hour after participants confirmed their “on-medication” status. A neurologist evaluated participants’ Unified Parkinson’s Disease Rating Scale (UPDRS) Part III (motor examination). Participants’ demographics and clinical characteristics were reported in the original paper [19].

### 2.2. Music Cues

Participants were provided with familiar and unfamiliar music cues. First, one song with a tempo in the range of 90–120 bpm (beat/min) was chosen from participants’ self-chosen familiar music selections, as this tempo was considered as an average walking tempo in people with PD [27]. Familiar music cues were also chosen based on the presence of salient beat rated by a trained musician. Second, one of two songs were selected as unfamiliar music cue. Participants with a walking cadence ≥ 113 steps/min at baseline were presented with American Author’s “What We Live For” (120 bpm) and participants with walking cadence < 113 steps/min at baseline were provided with Kyle Andrews’ “You Always Make Me Smile” (107 bpm). The tempo of all music cues was scanned by an audio-mixing software for later modifications to individual walking cadence. The volume of cues was set at 89 dB using a volume normalization software (MP3Gain Express 2.1.0, available at mp3gain.sourceforge.net, accessed on 25 July 2022). One song was chosen for each condition to minimize gait and listening trials to minimize potential fatigue in people with PD during data collection.

### 2.3. Gait Trials

The Ambulatory Parkinson’s Disease Monitoring system (128 Hz, APDM Inc., Portland, OR, USA) was used to record and analyze participants’ movements. Six non-invasive, wireless sensors, namely Opals^®^, were strapped on the participants’ feet, wrists, waist, and sternum, based on the Mobility Lab software package in the APDM system. Participants first completed a baseline trial, which entailed walking naturally in their comfortable pace for two minutes. After the baseline trial, individual walking cadence recorded during the baseline trial was checked through the APDM system. Then, the tempo of music cues was adjusted to individual walking cadence using a sound-editing software (Audacity 2.1.0; available at audacityteam.org, accessed on 25 July 2022).

After this, participants completed a total of four experimental walking trials in time with the beat of familiar and unfamiliar music cues (two trials for each), presented in a randomized order. Participants initiated walking when a music cue began and kept walking for approximately 2 min until the music cue randomly stopped. Music cues were randomly stopped to prevent participants from having expectations for the end of the walking trials. During the walking trials, participants were wearing a Bluetooth headphone that was connected to a laptop, through which music cues were played and outside noise was blocked.

After each walking trial, participants were instructed to take a break for 1 min which afforded the opportunity to disengage from a prior music cue. After the break, participants completed a backward counting task in which they counted loudly from 30 to 0 to facilitate the “wash-out” process. This method was found to be viable in an emotional memory recall paradigm [28] and musically-cued gait protocol in people with PD [18]. A 5 min break was given after the first session, during which participants listened to unfamiliar and familiar music again. Then, the second session followed, consisting of two gait trials with the same familiar and unfamiliar music cues (one trial for each), in a randomized order. All trials were performed by walking the outer perimeter (approximately 94 × 50 ft) of an indoor gym court.

### 2.4. Covariates

**Figure 1:**
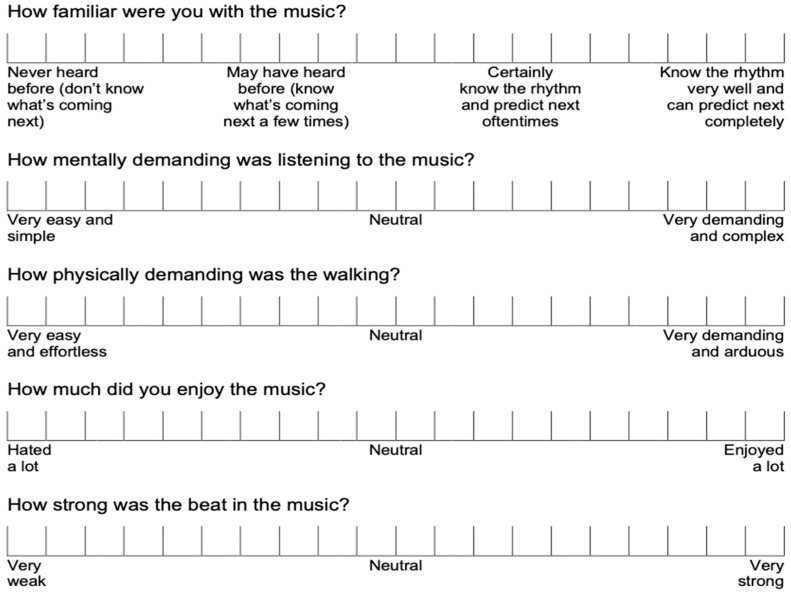
Visual analog scales for the perception of music cues.

Before the gait trials, participants completed Barcelona Music Reward Questionnaire (BMRQ, 20 items), a self-report measure of individual differences in music reward (i.e., how people experience reward in music-related activities) [29]. Based on the scoring algorithm, BMRQ scores were further processed into five factors (i.e., music seeking, emotion evocation, mood regulation, sensory-motor, and social reward) [29]. After each walking trial, participants indicated their perceived familiarity, enjoyment, cognitive demand, physical demand, and beats salience using 20-point visual analog scales (VAS). See Figure 1 for an overview of the scales used.

### 2.5. Data Analysis

The hypothesis was tested through multilevel modeling, which allowed for a detailed specification of fixed and random variations of gait parameters between and within subjects. The outcome variables of multilevel modeling were stride length and stride-to-stride time coefficient of variation (CV) as major indices of gait alterations in the prior study [19]. In our modeling, fixed effects were examined at two levels (level 2 and level 1). Level 2 fixed effects indicate the associations between individual variation in an independent variable and individual variation in an outcome variable across all participants. Level 1 fixed effects indicate associations of within-person variation in an independent variable and within-person variation in an outcome variable across all participants and occasions. Random effects represent individual differences in the level 1 fixed effects. Multilevel modeling enabled us to examine how familiarity, session, enjoyment, cognitive demand, beat salience, and/or music reward distinctively predict gait changes both within-persons (level 1) and between-persons (level 2). Within-person centering was used to calculate occasional deviations of an independent variable from its individual average (i.e., each of the occasion-varying raw values of an independent variable minus the individual average values of an independent variable across repeated occasions) in order to dissociate the level 1 effects of an independent variable from its effects at level 2 [30].

All models were estimated based on a maximum likelihood method to reach the highest statistical power and the least possibility of making a type I error [31,32]. The capacity of a model to predict gait changes was computed as an index of goodness of fit, assessed via the reduction in within- and between-person residual variances relative to a null model [33]. A decreased error of model prediction was estimated by the reduction in residual variances [32]. The number of units in our modeling (*n* = 20) met the required sample size to acquire sufficient power to detect multilevel effects [32]. A total of six steps of the modeling procedure were completed. Step 1 estimated the null model to determine the total variance to be explained and the goodness of fit to be improved by subsequent models. Step 2 and 3 estimated fixed and random effects of familiarity, session, and condition–session interaction on gait changes. Steps 4–8 estimated the effects of enjoyment, cognitive demand, physical demand, and beat salience. For each of these steps, the fixed level 2 effect, the fixed level 1 effect, and/or a random level 1 effect were estimated. Step 7 estimated the effects of music reward subfactors to examine the level 2 effects.

Variables with high collinearity (correlation coefficient higher than 0.50) were orthogonalized by creating a residual term in a regression model. Orthogonalized variables were (1) within-person variance of enjoyment that was not explained by within-person variance of familiarity, (2) within-person variance of beat salience that was not explained by within-person variance of enjoyment, and (3) between-person variance of cognitive demand that was not explained by between-person variance of beat salience. This method allowed us to examine the unique effects of the independent variables while controlling for multicollinearity. The SPSS Statistics 22.0 (IBM Corp, Chicago, IL, USA) software was used for all statistical analyses.

## 3. Results

In the null model, ICC were calculated as an index of level 1 (within-person) and level 2 (between-person) variance to be explained. For stride length changes, 39% of the overall variance was within-person and 61% was between-person variance. For stride time CV changes, 55% of the overall variance was within-person and 45% was between-person variance. Thus, the null model indicated a substantial amount of level 1 and level 2 variance that could be addressed by subsequent models in the next steps.

### 3.1. Cognitive and Affective Contributors to the Changes of Stride Length

Model fit results for each of added independent variables are summarized in Table 1. Results for stride length changes are described in the left side of Table 1. Each step of modeling resulted in decreases in -2LL. According to a nested χ^2^-test for examining model fit improvement, -2LL changes were significant when the fixed level 1 interaction effect of familiarity and session was added in step 4 (*p* < 0.05), and when the fixed level 2 and level 1 effects of enjoyment were added in step 5 (*p* < 0.05). The model fit change was minimal (*p* > 0.10) in step 6 (fixed level 2 and level 1 effects of cognitive demand) and step 7 (fixed level 2 and level 1 effects of beat salience). The model fit significantly improved when fixed level 2 effects of mood regulation and emotion evocation were added in step 8 (*p* = 0.05 and *p* < 0.05, respectively). In step 9, the final model retained independent variables that made significant changes in the model fit.

The final model in reduced form is summarized in Table 2. Significant level 1 effects were found in familiarity that had within-person associations with stride length changes, indicating overall smaller stride length during unfamiliar compared to familiar music. Familiarity significantly interacted with session at level 1, substantiating the expected effects of familiarity manipulation on gait changes. Among the covariates, significant level 1 effects were further found in enjoyment and beat salience that had positive associations with within-person variances in stride length changes across all people. These results indicate that walking with greater enjoyment and strong perceived beat were associated with increased stride length at the within-person level.

Significant level 2 effects were also found in individual mood regulation and emotion evocation, which showed positive associations with individual variances of stride length changes. These results indicate that people who use music more for mood regulation and emotion evocation showed greater stride length changes compared with those who use music less for mood regulation and emotion evocation. No random effects were found. The final model explained 43% of total within-person variance and 53% of total between-person variance.

### 3.2. Cognitive and Affective Contributors to the Changes of Stride Time Variability

Results for stride time CV changes are described in the right side of Table 1. Each step of modeling led to a reduction of -2LL. According to a nested χ^2^-test, -2LL changes were minimal (*p* > 0.10) in step 2 (fixed effect of familiarity), indicating that stride time variability was not affected by familiarity. A nested χ^2^-test indicated that model fit significantly improved when fixed level 1 effects of session and its interaction with familiarity were added in step 2 (*p* < 0.01 and *p* < 0.05, respectively), and when fixed level 2 and level 1 effects of cognitive demand were added in step 4 (*p* < 0.01). Model fit improvements were marginally significant when fixed level 2 and level 1 effects of enjoyment were added in step 3 (*p* = 0.054), fixed level 2 and level 1 effects of beat salience were added in step 5 (*p* = 0.073), and fixed level 2 effect of total music reward was added in step 6 (*p* = 0.099).

The final model, in an optimized form, is summarized in Table 2. Significant level 1 effects were found in familiarity, which had within-person associations with stride time CV changes, indicating greater stride time variability in unfamiliar music than familiar music. Familiarity significantly interacted with session at level 1, substantiating the predicted effects of the familiarity manipulation on stride time variability. Among the covariates, significant level 1 effects were further found in enjoyment, which were negatively associated with within-person variances of stride length changes across all people. This finding indicates that walking trials with greater enjoyment were associated with reduced stride time variability.

Significant level 2 effects were found in individual variances of cognitive demand and beat salience, which had significant associations with individual variances of stride time variability changes. Participants with lower cognitive demand of music listening and strong perception of musical beat had lower stride time variability, and individuals with higher cognitive demand and weak beat salience vice versa. No random effects were found. The final model explained 31% of total within-person variance and 61% of total between-person variance.

**Table 2:** Covariates predicting spatiotemporal gait changes.

	Stride Length Change					Stride Time CV Change				
	B	SE	df	t	*p*	B	SE	df	t	*p*
Fixed effects–independent variables and covariates										
Within-person										
Fam	−3.41	1.15	42.65	−2.97	<0.01	58.02	19.92	60	2.91	<0.01
Session	−0.55	0.34	50.65	−1.60	0.12	10.54	5.75	60	1.83	0.07
Fam × Session	0.56	0.22	42.62	2.61	<0.05	−12.47	3.74	60	−3.33	<0.01
Enjoyment	0.33	0.11	45.05	3.04	<0.01	−4.16	1.87	60	−2.23	<0.05
Beat salience	0.30	0.15	50.43	2.08	<0.05	6.21	2.90	60	1.52	0.13
Between-person										
Cognitive demand	-	-	-	-	-	7.79	1.74	20	4.47	<0.001
Beat salience	-	-	-	-	-	6.21	2.90	20	2.14	<0.05
Music reward	-	-	-	-	-	−1.82	1.02	20	−1.78	0.09
Mood regulation	0.29	0.12	18.39	2.55	<0.05	-	-	-	-	-
Emotion evocation	0.23	0.10	18.39	2.24	<0.05	-	-	-	-	-
Random effects–covariance parameter estimate										
Within-person										
Session	0.13	0.08		*z* = 1.68	0.09	-	-		-	-
Within pseudo *R^2^* = 0.43 Between pseudo *R^2^* = 0.53						Within pseudo *R^2^* = 0.65 Between pseudo *R^2^* = 0.13				

## 4. Discussion

The parent study of this paper demonstrated that enhanced familiarity with music cues had acute effects on increasing stride length, reducing stride-to-stride variability, increasing enjoyment and perceived beat salience, and reducing cognitive demand of walking with music in people with PD. By further analyzing the data set, this paper describes that the cognitive and affective factors as covariates are indeed associated with the familiarity-driven alterations of stride length and stride-to-stride variability in people with PD. Our findings are summarized as follows: (1) condition-varying perceived enjoyment and beat salience are positively associated with increased stride length; (2) participants with more music reward for mood regulation and emotion evocation show greater stride length changes compared with those with less music reward; (3) condition-varying perceived enjoyment is positively associated with decreases in stride-to-stride variability; and (4) participants with lower cognitive demand of walking with music cues and higher beat salience show lower stride-to-stride variability compared with those with higher cognitive demand and lower beat salience. These results provide behavioral evidence of independent and interactive influences of cognitive and affective responses to music cues on spatiotemporal gait parameters in people with PD.

Our findings support the proposed account of prior findings that reduced cognitive demand and increased enjoyment would be the underlying mechanisms of the effects of enhanced familiarity with music cues on increasing gait velocity, reducing stride time variability, and improving step–beat entrainment accuracy, thereby resulting in more effortless entrainment of walking to music cues [24]. A gait study showed that affective states interact with familiarity with music to influence stride pace and length [34]. Another gait study also demonstrated that expressive features of music cues provide additional relaxing and activating influences on movements compared with isochronous metronome cues that primarily entrain movement timing [35]. Studies on passive music listening have shown that enhanced familiarity with music by repeated listening led to higher ratings of pleasure and/or arousal [25,36]. While these prior findings and explanation were based on healthy adult populations, this study demonstrated that the proposed mechanism of affective engagement in rhythmic entrainment may be applicable to the people with PD. This finding agrees with the evidence that musical pleasure is related with the improvement of gait amplitude and dynamics in people with PD [18].

Our behavioral findings reflect the neurological bases from which they emerge in that neuroimaging data substantiate the influences of familiarity with music on both emotion and motor systems. A neuroimaging study showed that listening to familiar music led to greater activation of emotion-related limbic and paralimbic regions, as well as reward circuits, compared with listening to unfamiliar music [26]. Interestingly, the neuroimaging study also demonstrated that the motor cortex was more active when listening to liked music compared with disliked music [26]. Another neuroimaging study showed that pleasant responses to familiar music are accompanied by dopamine release in the reward circuits [37]. Dopaminergic activity in the reward circuits provides signals to implicitly motivate autonomic and spontaneous movements, which is diminished in people with PD and, thus, results in sluggish movement (bradykinesia) [38]. Our finding that entrainment-driven improvement of movement is associated with affective responses to music cues, as well as individual level of music reward, implies the potential role of dopaminergic activity in the reward circuits and concurrent activation of the motor system. This neural mechanism is a tentative assertion now and, thus, further investigation is necessary, especially in people with PD who have diminished dopaminergic activity in the brain.

Our findings also provide converging evidence of reciprocal relations between motor entrainment and affective responses to music in people with PD. Psychologists have described rhythmic entrainment as a core mechanism underlying musical emotion evocation [39,40,41,42]. This perspective may substantiate the predisposition of preverbal infants to engage in more rhythmic movement in response to music and drumbeats than non-rhythmic sounds (e.g., human speech) [14]. Interestingly, the duration of the infants’ rhythmic movement was associated with the display of positive affect (rated by the duration of smiles) [14]. Thus, it is also possible that enhanced familiarity with music cues facilitates entrainment, which then boosts the enjoyment of walking with music cues in people with PD.

Another interesting finding would be the association of cognitive demand with the alteration of stride-to-stride variability during musically cued walking in people with PD. Despite the cumulative evidence upholding the benefits of RAS for increasing gait pace in individuals with PD [6,7,8], its impact on gait variability has been inconsistently reported, and has mostly used metronome cues rather than music. In people with PD, for example, metronome cues delivered at 90% [10] and 100% [11] of baseline walking cadence decreased stride-to-stride time variability, whereas a metronome cue at 100% of baseline did not alter stride-to-stride variability [9]. Metronome cueing as a simple form of RAS can provide temporal reference and, thus, reduce attentional costs for initiating and executing steps, which entails entrainment and consequently decreases stride-to-stride variability in persons with PD [10]. However, the explicit role of music cues in altering stride-to-stride variability of the Parkinsonian gait has been largely uninvestigated and, therefore, more investigation is needed.

Walking to music cues would be more cognitively demanding than metronome cues due to the greater number of acoustic events and complicated rhythmic structures. Indeed, the synchronization performance of stepping to a music cue was found to be less accurate and more variable compared with stepping to a metronome cue, arguably due to the cognitive demand for beat perception [43]. Cognitive demand of entrainment to music cues could be more pronounced in people with PD, due to the diminished capacity for beat perception and rhythm discrimination [44]. Therefore, our finding that cognitive demand is positively associated with improving stride-to-stride variability during musically cued walking in people with PD is promising in that it informs the beneficial application of music cues for gait variability in people with PD. Additional research is needed to specify the impacts of music cues on gait variability in people with PD at a varying pace, and how such impacts can be differed by the amount of acoustic events and the complexity of the rhythmic structure.

## 5. Conclusions

Our study extends the knowledge base concerning how affective and cognitive attributes of music cues play a role in gait rehabilitation in people with PD. The current investigation yielded novel findings and promising directions for future research. Relative strengths and limitations of the investigation should be acknowledged to facilitate replication and extension. A notable strength of the study is the application of up-to-date statistical analysis techniques for identifying meaningful covariates of the effects of familiarity with music on stride length and stride time variability in people with PD. Linked with previous neurological evidence, the emerging body of literature implies that activation of the mesolimbic dopamine system via pleasant and familiar music cues can, in turn, activate or compensate for impaired motor circuits in people with PD. Our study also has some limitations. While the distinct roles of the covariates were addressed, the potential interactive effects of these covariates were not examined. Such interactions could be clarified by future studies with carefully controlled experimental conditions targeted to tease out these contributing factors. Future investigations also need to explore the unique benefits of affective–motor interactions and cognitive–motor interactions through music in people with PD. Moreover, the reported data were only captured during the gait trials with familiar and unfamiliar music cues and, therefore, there is no attempt to determine how long the acute improvements in gait would be retained, which would be vital information for clinicians. Future studies may benefit by specifying the retention effects of RAS on gait outcomes in different stages of PD.

## Figures and Tables

**Table 1:** Multi-step modeling procedure and model fit changes.

	Stride Length Change						Stride Time CV Change					
Models	-2LL	Δ-2LL	s2W	s2B	r2W	r2B	-2LL	Δ-2LL	s2W	s2B	r2W	r2B
(1) Null	409.96	-	6.00	9.34	-	-	841.39	-	1503.93	1503.93	-	-
(2) Fam added	407.55	2.42	5.77	9.40	0.04	−0.01	841.35	0.04	1502.97	1502.97	0.00	0.00
(3) Session added	402.22	5.33 *	4.91	7.55	0.18	0.19	832.43	8.93 **	1295.21	1295.21	0.14	−0.04
(4) Fam × Session added	397.24	4.98 *	4.44	7.60	0.26	0.19	826.56	5.86 *	1174.63	1174.63	0.22	−0.07
(5) Enjoyment added	391.10	11.12 **	3.90	7.79	0.35	0.17	820.73	5.84 *	1071.58	1071.58	0.29	−0.07
(6) Cog demand added	390.06	1.05	3.94	7.40	0.34	0.21	810.37	10.36 **	1068.33	1068.33	0.29	0.44
(7) Beat salience added	385.84	4.22	3.48	7.83	0.42	0.16	805.13	5.23	1029.46	1029.46	0.32	0.54
(8) Music reward added	377.69	8.15 *	3.39	4.27	0.43	0.54	800.52	4.61	1029.46	1029.46	0.32	0.68
(9) Reduced form	378.18	−0.49	3.43	4.43	0.43	0.53	803.20	−2.68	1031.70	1031.70	0.31	0.61

Abbreviations are as follows: CV, coefficients of variation; Fam, familiarity; -2LL, -2 log likelihood; Δ-2LL, change in -2LL relative to preceding model; s^2^W, an estimate of residual indicating unexplained within-person variance; s^2^B, an estimate of intercept indicating between-person variance; r^2^W, within-person pseudo R-squared, an estimate of within-person variance change from the null model, explained by fixed and random variables; r^2^B, within-person pseudo R-squared, an estimate of between-person variance change from the null model, explained by fixed and random variables; significant improvements in goodness of fit were indicated by * *p* < 0.05, ** *p* < 0.01.

## Data Availability

The data presented in this study are available on reasonable request from the corresponding author. The data are not publicly available due to their containing information that could compromise the privacy of research participants.

## References

[1] Thaut M.H., Hoemberg V., von Wild K. (2016). Handbook of Neurologic Music Therapy.

[2] Samii A., Nutt J.G., Ransom B.R. (2004). Parkinson’s disease. Lancet.

[3] Spaulding S.J., Barber B., Colby M., Cormack B., Mick T., Jenkins M.E. (2013). Cueing and Gait Improvement Among People with Parkinson’s Disease: A Meta-Analysis. Arch. Phys. Med. Rehabil..

[4] Lim I., van Wegen E., de Goede C., Deutekom M., Nieuwboer A., Willems A., Jones D., Rochester L., Kwakkel G. (2005). Effects of external rhythmical cueing on gait in patients with Parkinson’s disease: A systematic review. Clin. Rehabil..

[5] Rubinstein T.C., Giladi N., Hausdorff J.M. (2002). The power of cueing to circumvent dopamine deficits: A review of physical therapy treatment of gait disturbances in Parkinson’s disease. Mov. Disord..

[6] Ghai S., Ghai I., Schmitz G., Effenberg A.O. (2018). Effect of rhythmic auditory cueing on parkinsonian gait: A systematic review and meta-analysis. Sci. Rep..

[7] Nombela C., Hughes L.E., Owen A.M., Grahn J.A. (2013). Into the groove: Can rhythm influence Parkinson’s disease?. Neurosci. Biobehav. Rev..

[8] Rocha P.A., Porfírio G.M., Ferraz H.B., Trevisani V.F.M. (2014). Effects of external cues on gait parameters of Parkinson’s disease patients: A systematic review. Clin. Neurol. Neurosurg..

[9] Hausdorff J.M., Lowenthal J., Herman T., Gruendlinger L., Peretz C., Giladi N. (2007). Rhythmic auditory stimulation modulates gait variability in Parkinson’s disease. Eur. J. Neurosci..

[10] Baker K., Rochester L., Nieuwboer A. (2008). The effect of cues on gait variability—Reducing the attentional cost of walking in people with Parkinson’s disease. Parkinsonism Relat. Disord..

[11] Arias P., Cudeiro J. (2008). Effects of rhythmic sensory stimulation (auditory, visual) on gait in Parkinson’s disease patients. Exp. Brain Res..

[12] Zhang S., Liu D., Ye D., Li H., Chen F. (2017). Can music-based movement therapy improve motor dysfunction in patients with Parkinson’s disease? Systematic review and meta-analysis. Neurol. Sci..

[13] Forte R., Tocci N., De Vito G. (2021). The Impact of Exercise Intervention with Rhythmic Auditory Stimulation to Improve Gait and Mobility in Parkinson Disease: An Umbrella Review. Brain Sci..

[14] Zentner M., Eerola T. (2010). Rhythmic engagement with music in infancy. Proc. Natl. Acad. Sci. USA.

[15] Thaut M.H. (2015). The discovery of human auditory-motor entrainment and its role in the development of neurologic music therapy. Prog. Brain Res..

[16] Levitin D.J., Grahn J.A., London J. (2018). The Psychology of Music: Rhythm and Movement. Annu. Rev. Psychol..

[17] Damm L., Varoqui D., De Cock V.C., Dalla Bella S., Bardy B. (2020). Why do we move to the beat? A multi-scale approach, from physical principles to brain dynamics. Neurosci. Biobehav. Rev..

[18] Park K.S., Hass C.J., Patel B., Janelle C.M. (2020). Musical pleasure beneficially alters stride and arm swing amplitude during rhythmically-cued walking in people with Parkinson’s disease. Hum. Mov. Sci..

[19] Park K.S., Hass C.J., Janelle C.M. (2021). Familiarity with music influences stride amplitude and variability during rhythmically-cued walking in individuals with Parkinson’s disease. Gait Posture.

[20] de Bruin N., Doan J.B., Turnbull G., Suchowersky O., Bonfield S., Hu B., Brown L.A. (2010). Walking with Music Is a Safe and Viable Tool for Gait Training in Parkinson’s Disease: The Effect of a 13-Week Feasibility Study on Single and Dual Task Walking. Parkinson’s Dis..

[21] Thaut M.H., McIntosh G.C., Rice R.R., Miller R.A., Rathbun J., Brault J.M. (1996). Rhythmic auditory stimulation in gait training for Parkinson’s disease patients. Mov. Disord..

[22] McIntosh G.C., Brown S.H., Rice R.R., Thaut M.H. (1997). Rhythmic auditory-motor facilitation of gait patterns in patients with Parkinson’s disease. J. Neurol. Neurosurg. Psychiatry.

[23] Benoit C.-E., Dalla Bella S., Farrugia N., Obrig H., Mainka S., Kotz S.A. (2014). Musically Cued Gait-Training Improves Both Perceptual and Motor Timing in Parkinson’s Disease. Front. Hum. Neurosci..

[24] Leow L.A., Rinchon C., Grahn J. (2015). Familiarity with music increases walking speed in rhythmic auditory cuing. Ann. N. Y. Acad. Sci..

[25] van den Bosch I., Salimpoor V., Zatorre R.J. (2013). Familiarity mediates the relationship between emotional arousal and pleasure during music listening. Front. Hum. Neurosci..

[26] Pereira C.S., Teixeira J., Figueiredo P., Xavier J., Castro S.L., Brattico E. (2011). Music and Emotions in the Brain: Familiarity Matters. PLoS ONE.

[27] Hass C.J., Malczak P., Nocera J., Stegemöller E.L., Shukala A., Malaty I., Jacobson C.E., Okun M.S., McFarland N. (2012). Quantitative Normative Gait Data in a Large Cohort of Ambulatory Persons with Parkinson’s Disease. PLoS ONE.

[28] Fawver B., Hass C.J., Park K.D., Janelle C.M. (2014). Autobiographically recalled emotional states impact forward gait initiation as a function of motivational direction. Emotion.

[29] Mas-Herrero E., Marco-Pallares J., Lorenzo-Seva U., Zatorre R.J., Rodriguez-Fornells A. (2013). Individual Differences in Music Reward Experiences. Music Percept. Interdiscip. J..

[30] Singer J.D., Willett J.B., Singer J.D., Willett J.B. (2003). Treating TIME More Flexibly. Applied Longitudinal Data Analysis.

[31] Singer J.D., Willett J.B., Singer J.D., Willett J.B. (2003). Introducing the Multilevel Model for Change. Applied Longitudinal Data Analysis.

[32] Kreft I.G., de Leeuw J. (1998). Introducing Multilevel Modeling.

[33] Luke D.A. (2004). Multilevel Modeling.

[34] Park K.S., Hass C.J., Fawver B., Lee H., Janelle C.M. (2019). Emotional states influence forward gait during music listening based on familiarity with music selections. Hum. Mov. Sci..

[35] Leman M., Moelants D., Varewyck M., Styns F., van Noorden L., Martens J.-P. (2013). Activating and relaxing music entrains the speed of beat synchronized walking. PLoS ONE.

[36] Schellenberg E.G., Peretz I., Vieillard S. (2008). Liking for happy- and sad-sounding music: Effects of exposure. Cogn. Emot..

[37] Salimpoor V.N., Benovoy M., Larcher K., Dagher A., Zatorre R.J. (2011). Anatomically distinct dopamine release during anticipation and experience of peak emotion to music. Nat. Neurosci..

[38] Pietro M., Hristova A., Krakauer J.W. (2007). Why Don’t We Move Faster? Parkinson’s Disease, Movement Vigor, and Implicit Motivation. J. Neurosci..

[39] Juslin P.N., Liljeström S., Västfjäll D., Lundqvist L.O., Juslin P.N., Sloboda J.A. (2010). How does music evoke emotions? Exploring the underlying mechanisms. Handbook of Music and Emotion: Theory, Research, Applications.

[40] Scherer K.R., Coutinho E., Cochrane T., Fantini B., Scherer K.R. (2013). How music creates emotion: A multifactorial process approach. The Emotional Power of Music: Multidisciplinary Perspectives on Musical Arousal, Expression, and Social Control.

[41] Trost W., Vuilleumier P., Cochrane T., Fantini B., Scherer K.R. (2013). Rhythmic entrainment as a mechanism for emotion induction by music: A neurophysiological perspective. The Emotional Power of Music: Multidisciplinary Perspectives on Musical Arousal, Expression, and Social Control.

[42] Trost W., Labbé C., Grandjean D. (2017). Rhythmic entrainment as a musical affect induction mechanism. Neuropsychologia.

[43] Leow L.A., Parrott T., Grahn J.A. (2014). Individual differences in beat perception affect gait responses to low- and high-groove music. Front. Hum. Neurosci..

[44] Grahn J.A., Brett M. (2009). Impairment of beat-based rhythm discrimination in Parkinson’s disease. Cortex.

